# A Computed Tomography-Derived Radiomics Approach for Predicting Uncommon EGFR Mutation in Patients With NSCLC

**DOI:** 10.3389/fonc.2021.722106

**Published:** 2021-12-16

**Authors:** Wufei Chen, Yanqing Hua, Dingbiao Mao, Hao Wu, Mingyu Tan, Weiling Ma, Xuemei Huang, Jinjuan Lu, Cheng Li, Ming Li

**Affiliations:** Department of Radiology, Huadong Hospital, Fudan University, Shanghai, China

**Keywords:** NSCLC, computed tomography, uncommon EGFR, radiomics, nomogram

## Abstract

**Purpose:**

This study aims to develop a CT-based radiomics approach for identifying the uncommon epidermal growth factor receptor (EGFR) mutation in patients with non-small cell lung cancer (NSCLC).

**Methods:**

This study involved 223 NSCLC patients (107 with uncommon EGFR mutation-positive and 116 with uncommon EGFR mutation-negative). A total of 1,269 radiomics features were extracted from the non-contrast-enhanced CT images after image segmentation and preprocessing. Support vector machine algorithm was used for feature selection and model construction. Receiver operating characteristic curve analysis was applied to evaluate the performance of the radiomics signature, the clinicopathological model, and the integrated model. A nomogram was developed and evaluated by using the calibration curve and decision curve analysis.

**Results:**

The radiomics signature demonstrated a good performance for predicting the uncommon EGFR mutation in the training cohort (area under the curve, AUC = 0.802; 95% confidence interval, CI: 0.736–0.858) and was verified in the validation cohort (AUC = 0.791, 95% CI: 0.642–0.899). The integrated model combined radiomics signature with clinicopathological independent predictors exhibited an incremental performance compared with the radiomics signature or the clinicopathological model. A nomogram based on the integrated model was developed and showed good calibration (Hosmer–Lemeshow test, *P* = 0.92 in the training cohort and 0.608 in the validation cohort) and discrimination capacity (AUC of 0.816 in the training cohort and 0.795 in the validation cohort).

**Conclusion:**

Radiomics signature combined with the clinicopathological features can predict uncommon EGFR mutation in NSCLC patients.

## Introduction

Lung cancer is one of the leading causes of cancer-related deaths worldwide. Thereinto, non-small cell lung cancer (NSCLC) accounts for approximately 85% of all lung cancer cases (1). Over the past decade, the research of molecular targeted agents for NSCLC has made a great progress. The role of molecular targeted biomarkers in the process of oncotherapy has been further promoted (2). The epidermal growth factor receptor (EGFR) has been identified as the most common therapeutic biomarker for NSCLC. EGFR-TKI (tyrosine kinase inhibitor) treatment in EGFR mutation-activating patients has manifested superior progression-free survival benefits compared with standard chemotherapy (3). Of note is the fact that the therapeutic efficiency is closely related to the subtype of EGFR mutations. The mutation anchoring the uncommon site is thought to associate with poor outcomes, as it represents a higher heterogeneity (4, 5). Taking this into account, the accurate identification of uncommon EGFR mutation will play an essential role in the therapeutic decision-making of NSCLC patients.

At present, the acquisition of EGFR mutation status mainly depends on tissue biopsy. However, more than 50% of NSCLC patients get insufficient tissue in clinical practice (6). What is more, adverse events of percutaneous puncture, such as hemorrhage and pneumothorax, were reported in 17.1% among elderly patients (7). Thus, a non-invasive, convenient, and cost-effective alternative is desired (8).

Recently, radiomics is regarded to have a promising role for diagnostic support as it is non-invasive and has quantitative property to tumor heterogeneity. Previous studies demonstrated that radiomics signature could provide novel predictive indicators for the EGFR expression of NSCLC patients (9, 10). However, the study of predicting the subtype of EGFR mutation with radiomics analysis has been rarely reported.

This study aimed to evaluate the feasibility of radiomics approach to predict the uncommon EGFR mutation in NSCLC patients. We expect that this approach will become an alternative for optimizing the treatment for NSCLC patients.

## Materials and Methods

### Data of Patients

This study was approved by our institutional review board, and the informed consent requirement for using desensitized data was waived. Consecutive patients with pathologically confirmed NSCLC from January 2016 to December 2020 were retrospectively analyzed. CT images and clinicopathological data were collected from the picture archiving and communication system (PACS) and the hospital information system in our institution. EGFR mutations of wild, common, and uncommon type were examined with human gene mutation detection kit (AmoyDx, China) *via* real-time polymerase chain reaction (PCR)-based assay and confirmed through direct sequencing.

The inclusion criteria were as follows: (a) no chemotherapy, radiotherapy, or targeted therapy before CT acquisition and PCR analysis; (b) cases with radiomics features that could be effectively extracted from the CT images; and (c) available clinicopathological data.

Finally, a total of 223 patients (107 with uncommon EGFR mutation, 73 with common EGFR mutation, and 43 with wild type) were enrolled in this study. The cases were randomly divided into the training cohort and the validation cohort at a ratio of 4:1.

### Image Acquisition and Segmentation

All patients in this study underwent non-contrast-enhanced CT that covered the entire thorax. The scanning parameters are detailed in **Supplementary Table S2**. Images in DICOM format were derived from PACS in our institution. We used a commercially available segmentation software (Yizhun CIPS, version 4.0; http://www.yizhun-ai.com/Content/477572.html) and its lung tumor analysis tool as our image segmentation platform. The regions of interest (ROI) were delineated manually by two radiologists with more than 6 and 13 years of experience in chest CT interpretation with reference to the mediastinum and lung window, respectively. Both radiologists were blinded to the clinicopathological information and EGFR mutation status. Another radiologist with 7 years of experience independently segmented a random set of 20 nodules to assess the inter-observer reproducibility.

### Radiomic Feature Extraction and Selection

The radiomics feature extraction in this study was performed with pyRadiomics (https://doi.org/10.1158/0008-5472.CAN-17-0339). Before the feature extraction, we used the nearest neighbor interpolation algorithm to resample the voxel into an isotropic distribution of 1 × 1 × 1 mm. Gaussian filter was used to modify the outlier value of voxel to reduce the photon noise influence on the radiomics features. A total of 1,269 radiomics features, which made up a mineable database for excavating the phenotype biomarker of the uncommon EGFR mutation, were extracted from the ROI. The definition of these radiomics features is available at http://pyradiomics.readthedocs.io/en/latest/features.html.

Inter-class correlation coefficient (ICC) was used to assess the inter-observer reproducibility of the extractive features. An ICC >0.75 was considered a good agreement. Stable and reproducible features were entered in the process of feature selection. Maximal relevance and minimal redundancy was used to reduce the redundant features.

### Prediction Model Construction

The support vector machine (SVM), which is suitable for a small sample set, was adopted to construct the radiomics model in this study. The key features and their corresponding weight were calculated and screened out in the training cohort. Then, a radiomic score (Rad-score) was built by the weighted linear combination of all key features.

To explore the optimal model, another model based on clinicopathological features, including sex, age, smoking history, tumor grade, tumor biomarkers, stage, and Eastern Cooperative Oncology Group Performance Status (ECOG PS), was simultaneously built with multivariate logistic regression analysis. An integrated model, which included the Rad-score and the clinicopathological independent predictors, was also constructed.

Receiver operating characteristic (ROC) curve analysis was used to evaluate the predictive performance of each model. Then, the superior model was chosen to draw a nomogram for evaluating the clinical application. The calibration curve was plotted to explore the predictive accuracy of the nomogram. Decision curve analysis (DCA) was implemented to quantify the net benefits.

### Statistical Analysis

Statistical analysis was performed using R software (version 3.6.2; R Foundation for Statistical Computing, Vienna, Austria; http://www.Rproject.org) and SPSS 21.0 (IBM, Chicago, IL, USA). R packages of “e1071”, “rms”, and “rmda” were implanted to execute the algorithm of SVM, nomogram, and DCA, respectively. Multivariate binary logistic regression was done with input parameter strategy. Independent *t*-test was used for the continuous variables, and chi-square test or Fisher’s exact test was used for the categorical variables. All statistical tests were two-tailed, and *P <*0.05 indicated a significant difference.

## Results

### Performance of the Clinicopathological Model

Among the full cohort, 107 patients were tested as uncommon EGFR mutation. The number of 20-INS, G719X, L861Q, S768I, and mixed was 23 (21.5), 33 (30.8), 26 (24.3), 11 (10.3), and 14 (13.1), respectively. No patients in this study had more than 2 exon mutations. Most patients exhibited a histological type of lung adenocarcinoma. According to disease-free survival (11), we incorporated the histological subtypes of non-mucinous lepidic predominant, acinar predominant, and papillary predominant adenocarcinoma into the low/intermediate-grade cohort and other subtypes, including solid predominant, micropapillary predominant invasive mucinous adenocarcinoma, and SCC, into the high-grade cohort. The EGFR mutation and histological subtype are detailed in **Supplementary Table S1**.

The clinicopathological features of the training and the validation cohorts are summarized in **Table 1**. Univariate analysis indicated that there was no significant difference in age, sex, smoking status, or tumor markers of NSE, CA125, SCC, CY21-1, stage, and ECOG PS between uncommon EGFR mutation-positive and uncommon EGFR mutation-negative (*P* > 0.05). Within the two cohorts, the uncommon EGFR mutation-positive showed a significant difference in serum carcinoembryonic antigen (CEA) and tumor grade (*P* < 0.05). Accordingly, these two features were selected to establish a clinicopathological model with multivariate logistic regression analysis. The ROC curves for the clinicopathological model showed an acceptable performance (AUC of 0.665, 95% CI: 0.5995–0.727, sensitivity 63.55%, and specificity 62.93%).

**Table 1:** Clinicopathological data of patients in the training and validation cohorts.

Variable	Training cohort			Validation cohort			*P*
	Uncommon EGFR (+)	Uncommon EGFR (-)	*P*	Uncommon EGFR (+)	Uncommon EGFR (-)	*P*	
Age (mean ± SD)	64.93 ± 10.07	63.29 ± 9.89	0.273	65.47 ± 9.14	64.82 ± 13.7	0.856	0.543
Sex, *n* (%)			0.270			0.098	0.064
Male	45 (52.3)	41 (44.1)		16 (76.2)	12 (52.2)		
Female	41 (47.7)	52 (55.9)		5 (23.8)	11 (47.8)		
Smoking status, *n* (%)			0.879			0.063	0.822
Smoker	7 (8.1)	7 (7.5)		18 (85.7)	23 (100)		
Never smoker	79 (91.9)	86 (92.5)		3 (14.3)	0 (0)		
Grade, *n* (%)			**0.036**			**0.032**	0.137
Low/intermediate	62 (72.1)	79 (84.9)		11 (52.4)	19 (82.6)		
High	24 (27.9)	14 (15.1)		10 (47.6)	4 (17.4)		
Tumor marker (mean ± SD)							
CEA	7.63 ± 6.13	5.78 ± 5.49	**0.035**	8.53 ± 8.18	4.53 ± 4.20	**0.045**	0.823
NSE	2.87 ± 2.27	3.03 ± 2.99	0.695	2.46 ± 1.38	3.75 ± 3.11	0.087	0.686
CA125	9.49 ± 8.39	9.63 ± 6.18	0.898	8.67 ± 11.51	6.47 ± 13.48	0.567	0.157
SCC	0.69 ± 1.29	0.81 ± 1.23	0.506	0.92 ± 1.7	0.53 ± 0.28	0.294	0.848
CY21-1	3.61 ± 9.03	3.40 ± 3.65	0.834	3.52 ± 2.59	2.06 ± 2.34	0.056	0.475
Stage, *n* (%)			0.060			0.216	0.266
I	52 (60.5)	58 (62.4)		11 (52.4)	15 (65.2)		
II	9 (10.5)	11 (11.8)		1 (4.8)	1 (4.3)		
III	10 (11.6)	2 (2.2)		0 (0)	2 (8.7)		
IV	15 (17.4)	22 (23.7)		9 (42.9)	5 (21.7)		
ECOG PS, *n* (%)			0.368			0.594	0.580
0	29 (33.7)	34 (36.6)		6 (28.6)	6 (26.1)		
1	31 (36.0)	33 (35.5)		9 (42.9)	8 (34.8)		
2	18 (20.9)	23 (24.7)		5 (23.8)	5 (21.7)		
3	8 (9.3)	3 (3.2)		1 (4.8)	4 (17.4)		
Rad_score (mean ± SD)	0.55 ± 0.68	-0.29 ± 0.68	**0.001**	0.40 ± 0.84	-0.48 ± 0.68	**0.001**	0.970

Bold values: P < 0.05.

### Performance of the Radiomics Model

**Figure 1:**
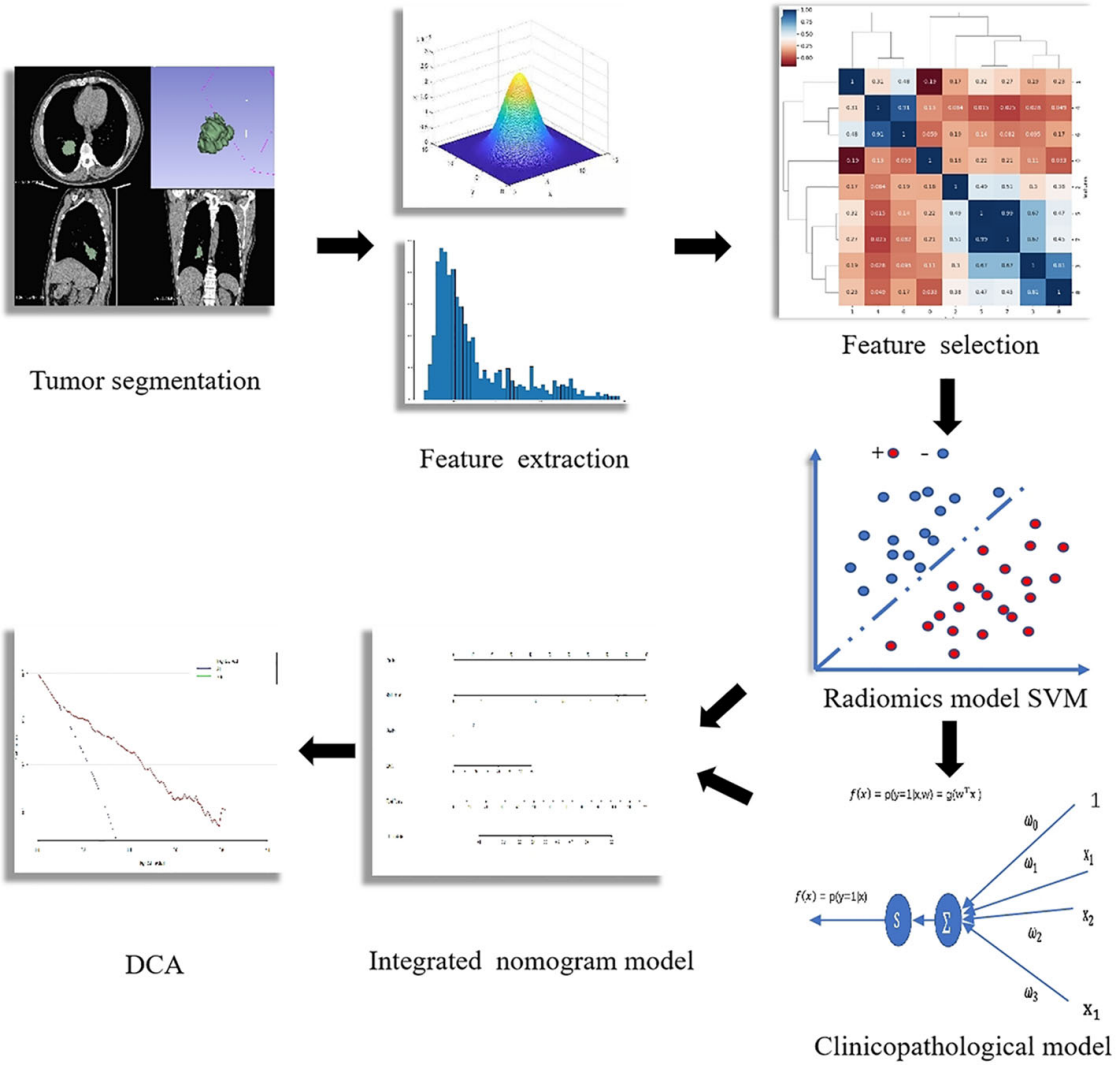
Workflow of the radiomics analysis.

The workflow of radiomics analysis is indicated in **Figure 1**. A total of 1,018 features with ICCs >0.75 were reserved according to the re-segmentation data. After SVM analysis, 10 robust radiomics features, which were associated with an uncommon EGFR mutation, remained in the training cohort. The detailed formula of the Rad-score is shown in the **Supplementary Material**. The ROC curve for the radiomics signature showed a good performance in the training cohort (AUC = 0.802; 95% CI: 0.736–0.858; sensitivity, 82.56%; and specificity, 78.49%) and was then verified in the validation cohort (AUC = 0.791; 95% CI: 0.642–0.899; sensitivity, 61.90%; and specificity, 91.30%).

### Performance of the Integrated Model

Subsequently, we established an integrated model with Rad-score, serum CEA, and the tumor grade. According to the multivariable logistic regression analysis, only Rad-score was independently associated with the uncommon EGFR mutation in the training cohort. The corresponding regression equation was as follows:


logit(p)=−0.417+1.601×Rad−score+0.058×CEA−0.334×grade.

The integrated model showed an incremental performance in the training cohort (AUC of 0.816; 95% CI: 0.751–0.870; sensitivity, 86.05%; and specificity, 70.97%) and AUC of 0.795 (95% CI: 0.646–0.902; sensitivity, 66.67%; and specificity, 91.3%) in the validation cohort.

**Figure 2:**
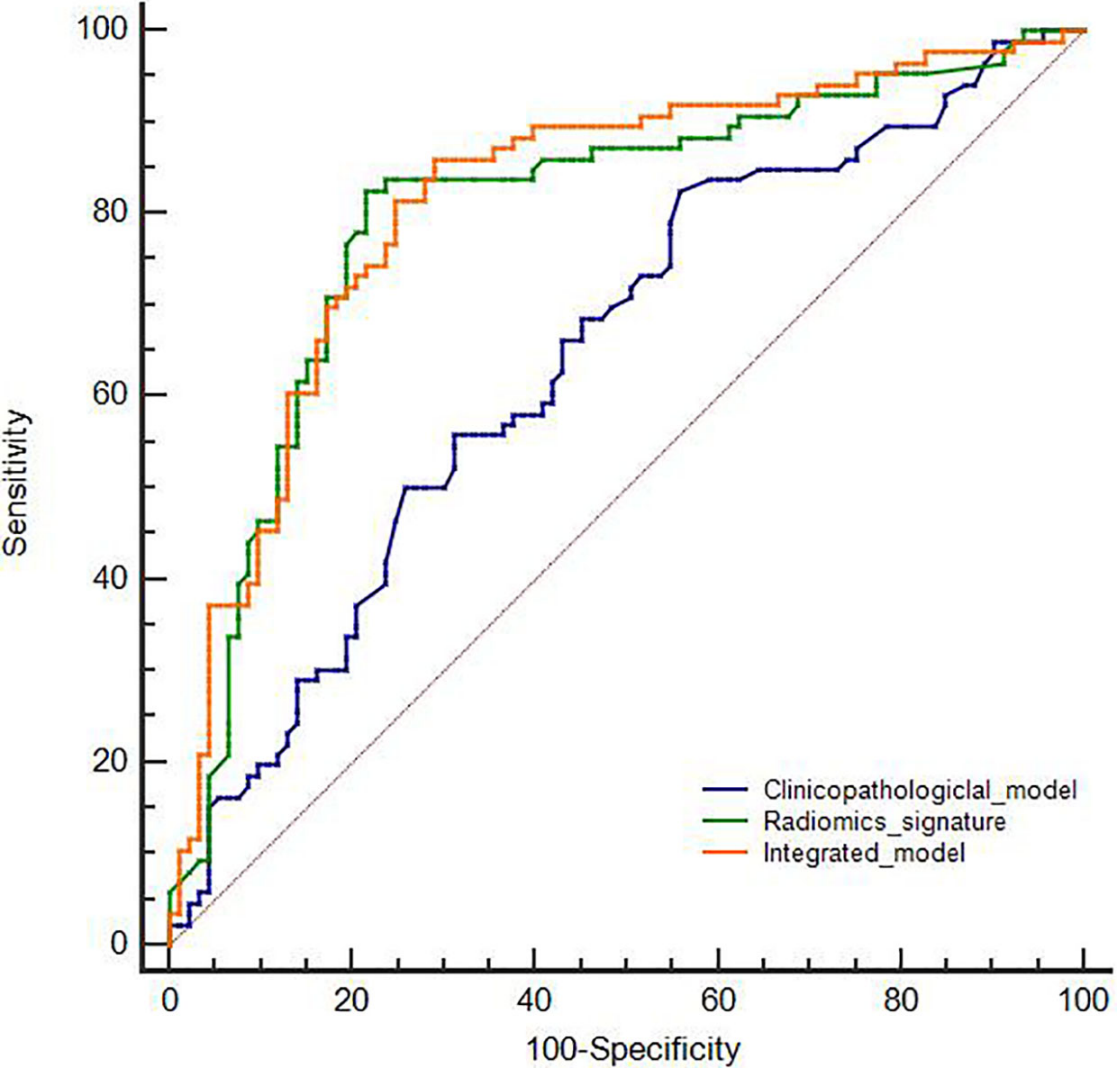
Receiver operating characteristic curves of the developed models in the training cohort.

The comparison of the three developed models is shown in **Figure 2**. According to the DeLong test, both the radiomics signature and the integrated model were superior to the clinicopathological model (*P* < 0.05). However, no statistical difference was found between the radiomics signature and the integrated model (*P* > 0.05).

### Nomogram Construction

**Figure 3:**
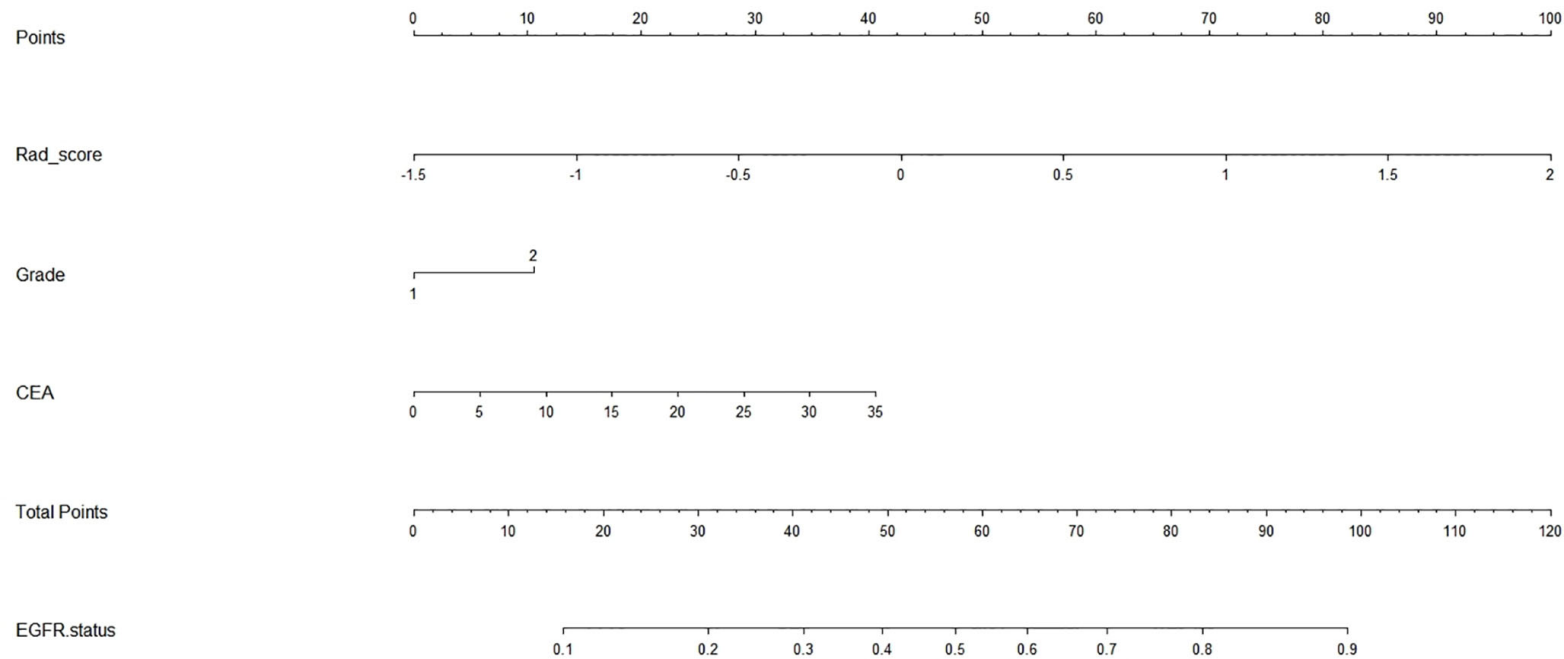
Radiomics nomogram. The nomogram incorporated the radiomics signature with serum carcinoembryonic antigen and tumor grade.

**Figure 4:**
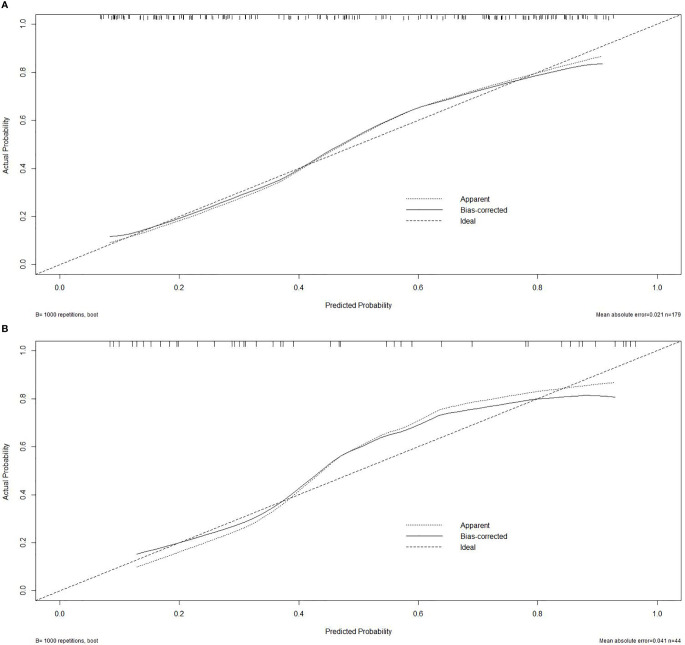
Calibration curve. **(A)** Calibration curve of the nomogram in the training cohort. **(B)** Calibration curve in the validation cohort.

To visualize the potential application of the developed model, a nomogram based on the integrated model was delineated (seen in **Figure 3**). The Hosmer–Lemeshow test showed no significant statistical difference between calibration curves and ideal curves both in the training cohort (*P* = 0.92) and in the validation cohort (*P* = 0.608). The calibration curve of the nomogram for the probability of the uncommon EGFR mutations demonstrated a good agreement (shown in **Figure 4**).

**Figure 5:**
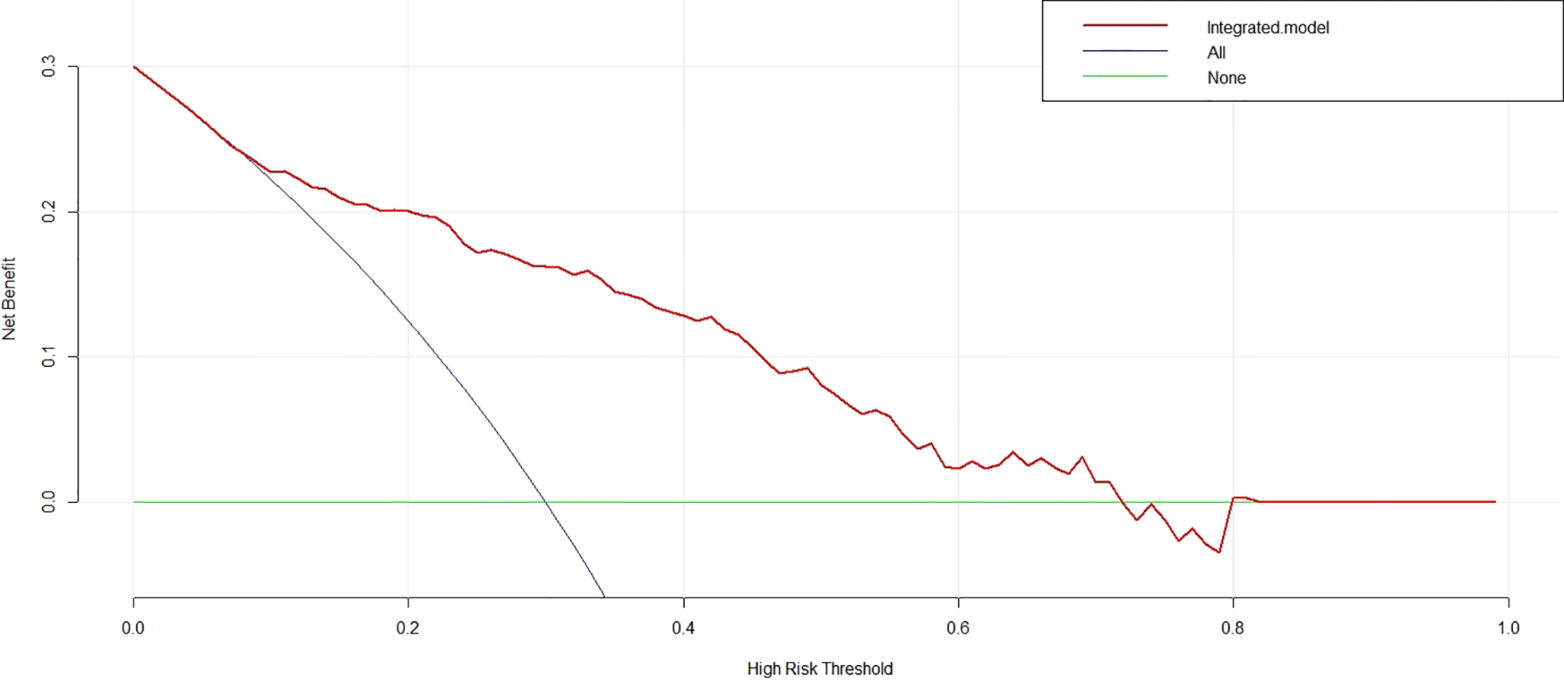
Decision curve analysis for the nomogram. With the threshold probabilities >10%, using the nomogram to predict the uncommon epidermal growth factor receptor status added more benefit than using the treat-all scheme or the treat-none scheme.

DCA was performed for the nomogram. As shown in **Figure 5** (red line), using the nomogram model to predict the uncommon EGFR mutation added more benefit than using the treat-all scheme or the treat-none scheme with the threshold probabilities >10%.

## Discussion

In this study, we developed a radiomics signature for non-invasive predicting of the uncommon EGFR mutation in NSCLC patients. The radiomics signature demonstrated good performance both in the training cohort (AUC = 0.802) and in the validation cohort (AUC = 0.791). We subsequently combined the radiomics signature with the clinicopathological independent predictors to construct an integrated model. The integrated model achieved an incremental performance with an AUC of 0.816 in the training cohort and 0.795 in the validation cohort. The nomogram based on the integrated model demonstrated an easy-to-use value with a good agreement on the calibration curve. When the DCA threshold probabilities >10%, using the nomogram obtained more benefit than using the treat-all scheme or the treat-none scheme.

Our results are in line with previous studies. Yip et al. (12) demonstrated that radiomics signature could successfully identify the EGFR-activating mutation in lung adenocarcinoma patients. Mei et al. (13) obtained moderate diagnostic performance in assessing the correlation between the radiomics features and EGFR exon 19 or 21 mutations of lung adenocarcinoma. To the best of our knowledge, this study is one of the firsts to evaluate the feasibility of radiomics features in predicting the uncommon EGFR mutation of NSCLC. Our result is reasonable, such that NSCLC with uncommon EGFR mutation represents a higher heterogeneous subgroup (14, 15), in which the heterogeneity is closely associated with radiomics phenotypes. We believe that our radiomics signature could provide clinical feasibility for identifying the uncommon EGFR mutation. In consideration of the different mechanisms of resistance (16, 17), our future study will dedicate to investigating the radiomics changes correlating with the subtype of uncommon EGFR mutation such as C797S and T790M.

Clinicopathological factors have been recognized as an important indicator of EGFR mutation (18, 19). Previous researches (20) have demonstrated that the combination of clinicopathological factors and radiomics signature could complement the information and improve the model prediction ability for EGFR mutation. In this study, we found that serum CEA and tumor grade were potentially associated with uncommon EGFR mutation, whereas no significant correspondence was found in stage and ECOG PS, which had been proved to have independent prognostic value for NSCLC patients in previous studies. One explanation may be that both of these factors represent the general status of tumor and patients instead of intratumoral conditions. Our integrated model exhibited an incremental performance, but no significant difference was found between the integrated model and the radiomics signature, probably because the serum CEA and tumor grade are sensitive to the poor differentiation of tumor but insensitive to tumor heterogeneity.

We next constructed a radiomics nomogram based on the integrated model. As seen in **Figure 3**, the nomogram is expected to become a supporting tool for clinicians following their experience and judgment. It is worth noting that another minimally invasive approach of liquid biopsy has been receiving more and more attention in recent years (21, 22). Both radiomics and liquid biopsy could provide objective, comprehensive, and virtually real-time information for EGFR testing, but drawbacks in using them in isolation make them complementary. Firstly, ctDNA, as an example of liquid biopsy, is less sensitive and specific than ideal (23). It is unclear whether the sample could represent all genetic clones, such that ctDNA accounts for only 0.02 to 0.1% of the total DNA circulating. To make up for that, we can use radiomics to provide a full-field analysis and refine the liquid biopsy results. Besides this, no clear biological explanation has been made for radiomics. Liquid biopsy may help to decode the biological significance of tumor information. Lastly, both the radiomics and molecular protocols need to be standardized. Extremely sensitive analytical instruments are needed. In future articles, we plan to make a combination of these two data to improve the credibility of the results.

Nevertheless, there are remaining limitations to this study. Firstly, this was a retrospective study and performed in a single center. Selection bias in patients was inevitable. Secondly, the sample size of the entire cohort was relatively small. Larger-sample-size studies are needed to further validate the reliability of the model. Lastly, the reconstruction kernel and scanner parameters of different CT vendors may have affected the stability of the radiomics features. In future investigations, a multicenter and prospective study with standardized CT scanning protocol is warranted to improve the stratification of uncommon EGFR mutation.

## Conclusion

NSCLC with uncommon EGFR mutation represents a highly heterogeneous entity, which exhibits resistant biological characteristics when treated with EGFR-TKI. The radiomics approach combined with clinicopathological information could effectively identify the uncommon EGFR mutation and help clinicians to optimize relevant therapeutic strategies.

## Data Availability Statement

The raw data supporting the conclusions of this article will be made available by the authors without undue reservation.

## Author Contributions

YQH and ML designed the study, MYT, WLM, XMH, CL, and JJL collected the data, HW was added to assess the interobserver reproducibility of radiomics feature extraction, WFC performed the data analyses, DBM and WFC critically revised the manuscripts, YQH supervised the study, and WFC wrote the manuscript. YQH and ML made an equal contribution to this article. All authors contributed to the article and approved the submitted version.

## Funding

This study was funded by the National Natural Science Foundation of China (grant no.: 61976238), “Future Star” of famous doctors’ training plan of Fudan University, the National Key Research and Development Program of China (grant no.: 2017YFC0112800 to Peijun Wang and grant no.: 2017YFC0112905 to Jinlong Shi), and the Medical Imaging Key Program of Wise Information Technology of 120, Health Commission of Shanghai (grant no.: 2018ZHYL0103 to ML).

## Conflict of Interest

The authors declare that the research was conducted in the absence of any commercial or financial relationships that could be construed as a potential conflict of interest.

## Publisher’s Note

All claims expressed in this article are solely those of the authors and do not necessarily represent those of their affiliated organizations, or those of the publisher, the editors and the reviewers. Any product that may be evaluated in this article, or claim that may be made by its manufacturer, is not guaranteed or endorsed by the publisher.
