# Supplementary material for: A Computed Tomography-Derived Radiomics Approach for Predicting Uncommon EGFR Mutation in Patients With NSCLC
Source: Front Oncol. 2021 Dec 16;11:722106. doi: 10.3389/fonc.2021.722106 (PMC8716946; doi:10.3389/fonc.2021.722106)
Supplement: Supplementary file 1 [file DataSheet_1.pdf]

TABLE S-1 | histological subtypes and EGFR mutation status of patients in the training and validation cohorts

	Training cohort			Validation cohort		
	Uncommon EGFR(+)	Uncommon EGFR(-)	p	Uncommon EGFR(+)	Uncommon EGFR(-)	p
Histological subtype (%)			0.074			0.051
LPA	8(9.3)	6(6.5)		1(4.8)	-	
APA	46(53.5)	55(59.1)		9(42.9)	15(65.2)	
PPA	8(9.3)	18(19.4)		1(4.8)	4(17.4)	
SPA	13(15.1)	5(5.4)		4(19.0)	-	
MPA	6(7.0)	5(5.4)		3(14.3)	-	
IMA	2(2.3)	3(3.2)		2(9.5)	1(4.3)	
SCC	3(3.5)	1(1.1)		1(4.8)	3(13.0)	
EGFR mutation (%)						
20-INS	17(19.8)	-		6(28.6)	-	
G719X	27(31.4)	-		6(28.6)	-	
L861Q	21(24.4)	-		5(23.8)	-	
S768I	10(11.6)	-		1(4.8)	-	
mixed	11(12.8)	-		3(14.2)	-	
19Del	-	24(25.8)		-	8(34.8)	
L858R	-	33(35.5)		-	8(34.8)	
wild-type	-	36(38.7)		-	7(30.4)	

Abbreviations: LPA:lepidic predominant adenocarcinomas, APA:acinar predominant adenocarcinoma; PPA:papillary predominant adenocarcinoma; MPA, micropapillary predominant adenocarcinoma; SPA,Solid predominant adenocarcinoma; IMA: invasive mucinous adenocarcinoma; SCC:Squamous cell carcinoma

TABLE S-2 | CT scanning parameters

parameters	GE Discovery CT750 HD	Somatom Definition Flash
Tube voltage（kVp）	120	120
Tube current(mAs)	200	110
Pitch	0.984:1	1.0
Collimation(mm)	0.625*64	0.6*64
Rotation time(s/rot)	0.5	0.33
SFOV(cm)	50	50
Slice thickness of reconstruction(mm)	1.25	1
Slice interval of reconstruction(mm)	1.25	1
Reconstruction algorithm	STND	Medium sharp

Rad_Score =

-1.917*original_gldm_DependenceEntropy+0.559*original_glszm_SizeZoneNonUniformityNormalized+0.496*original_glcm_JointAverage+0.399*original_glcm_MaximumProbability+0.381*original_glszm_SmallAreaEmphasis+0.337*original_glrlm_ShortRunHighGrayLevelEmphasis+0.280*wavelet-LHL_glcm_Autocorrelation+0.254*original_gldm_DependenceVariance+0.229*original_glcm_JointEnergy+0.170*original_gldm_LargeDependenceHighGrayLevelEmphasis-0.172
